# Developing the Patient Health Questionnaire-8 for a greater impact on the quality of life of patients with functional dyspepsia compared to Somatic Symptom Scale-8

**DOI:** 10.1186/s12876-020-01508-4

**Published:** 2020-10-28

**Authors:** Chaoqun Yuan, Guizhen Yong, Xi Wang, Ting Xie, Chunyan Wang, Yuan Yuan, Guobin He

**Affiliations:** 1 Department of Gastroenterology, Affiliated Hospital of North Sichuan Medical College, No. 67 Wenhua Road, Shunqing, Nanchong, 637000 Sichuan China; 2 Department of Nursing, North Sichuan Medical College, No. 234 Fujiang Road, Shunqing, Nanchong, 637000 Sichuan China

**Keywords:** Patient health questionnaire-8, Functional dyspepsia, Somatization, Somatic Symptom Scale-8, Reliability, Validity, Quality of life

## Abstract

**Background:**

To develop the Patient Health Questionnaire-8 (PHQ-8) as a more reliable approach than the Somatic Symptom Scale-8 (SSS-8), evaluating somatization which might be a critical factor influencing the quality of life (QoL) in patients with functional dyspepsia (FD). Also, the effects of somatization on QoL of FD patients were assessed by these two approaches.

**Methods:**

Herein, 612 FD patients completed a questionnaire involving 25 items. 8/25 items were selected to develop the PHQ-8 by four methods of discrete degree, correlation coefficient, factor analysis, and Cronbach’s α coefficient. Reliability and validity of the PHQ-8 and the SSS-8 were compared by principal component and confirmatory factor analyses. The effects of somatization, depression, and anxiety on the Nepean Dyspepsia Index (NDI) for QoL were explored by Pearson’s correlation coefficient and linear regression analysis.

**Results:**

The Cronbach’s α coefficient for the PHQ-8 and the SSS-8 was 0.601 and 0.553, respectively, and the cumulative contribution rate of three extracted factors for the developed PHQ-8 and SSS-8 was 55.103% and 51.666%, respectively. Somatization evaluated by the PHQ-8 (r = 0.309, *P* < 0.001) and the SSS-8 (r = 0.281, *P* < 0.001) was found to be correlated to NDI. The model used for the PHQ-8 showed that the values of goodness-of-fit index (GFI) and adjusted GFI (AGFI) were 0.984 and 0.967, respectively, which indicated that the model fitted well. Linear regression analysis unveiled that somatization (β = 0.270, *P* < 0.001), anxiety (β = 0.163, *P* < 0.001), and depression (β = 0.136, *P* = 0.003) assessed by the PHQ-8 were correlated to NDI. In addition, somatization (β = 0.250, *P* < 0.001), anxiety (β = 0.156, *P* < 0.001), and depression (β = 0.155, *P* = 0.001) evaluated by the SSS-8 were correlated to NDI.

**Conclusions:**

PHQ-8 showed a superior reliability and validity, and somatization assessed by the developed PHQ-8 showed a greater influence on the QoL of FD patients as compared to the SSS-8. Our findings suggested that the developed PHQ-8 may show improvement in a reliable assessment of the effects of somatization on FD patients in lieu of the SSS-8.

## Background

### Pathogenesis and subtypes of functional dyspepsia

Functional dyspepsia (FD) is characterized by bothersome epigastric pain or burning, postprandial fullness, or early satiation without evidence of structural disease. According to the statistics, the prevalence of FD is as high as 20–30% [1, 2]. The underlying pathogenesis includes diverse mechanisms, such as infectious causes represented by Helicobacter pylori (*H. pylori*) [1–6], diet factor [1, 7, 8], gastric acid [1, 9, 10], delayed gastric emptying, impaired proximal gastric accommodation [1, 11–13], visceral hypersensitivity [1, 14–16], duodenal inflammation [1, 17, 18], genetic factors [19, 20], and psychosocial factors (such as anxiety, depression, and stress) [19–25]. Moreover, these factors may interact with each other under the participation of brain-gut axis [1, 3, 15]. Thus, FD is a disorder of gut-brain interaction and classified into three subtypes based on Rome IV criteria: (1) epigastric pain syndrome (EPS): upper abdominal pain and/or burning discomfort of upper abdomen; (2) postprandial distress syndrome (PDS): postprandial fullness and early satiety; (3) the overlapped group of EPS and PDS [2].

### FD patients with common somatization symptoms

In addition, FD patients often have dizziness, back pain, sleep disorders, fatigue [26], and other symptoms of digestive system that cannot be explained by biochemical and structural abnormalities. Clinically, these symptoms are known as somatization symptoms [27, 28]. Somatization is defined as a chronic mental disorder characterized by the presence of one or more frequently changing somatic symptoms, involving multiple systems and organs of the body [29]. Those symptoms often induce patients’ incorrect understanding or excessive attention, imposing a huge economic burden on the society [30]. Somatization can coexist with other medical disorders, such as anxiety and depression, rendering them complex and changeable [31]. Additionally, it affects the severity of dyspepsia and the quality of life (QoL) of FD patients [32, 33]. Somatization plays a more significant role in dyspepsia symptom severity (DSS) as compared to that in gastric sensitivity, anxiety, and depression in FD patients [21]. It is an independent risk factor for impaired QoL of FD patients, and a 5-year follow-up study demonstrated that proximal gastric accommodation, gastric emptying, and *H. pylori* infection were not found as risk factors [33]. Therefore, assessment of somatization is highly essential for studying FD patients.

### Limitations of questionnaires for somatization

The symptoms of somatization disorder were widely assessed by the Patient Health Questionnaire-15 (PHQ-15) developed by Kroenke et al. [34]. However, PHQ-15 includes a number of items overlapped with gastrointestinal symptoms in FD patients. Therefore, symptoms of somatization disorder have been investigated by the Patient Health Questionnaire-12 (PHQ-12) with the removal of three gastrointestinal symptoms, while both PHQ-12 and PHQ-15 had dysmenorrhea or other items related to menstrual discomfort, making the gender score different [34–36].

On the other hand, symptoms of somatization disorder were evaluated by the Somatic Symptom Scale-8 (SSS-8) with satisfactory reliability and validity developed in the field of the Diagnostic and Statistical Manual of Mental Disorders (Fifth Edition) (DSM-5) [37–39]. Therefore, it was previously recommended to be a replacement for the PHQ-15 [40]. However, the SSS-8 is not a FD-specific scale for evaluating symptoms of somatization disorder, in which the relevant items may overlap with gastrointestinal symptoms. Subsequently, we developed the Patient Health Questionnaire-8 (PHQ-8) for assessing FD patients with symptoms of somatization disorder by adapting SSS-8, and confirmed its reliability and validity. The PHQ-8 had a greater influence on the QoL of FD patients than the SSS-8.

## Patients and methods

### Patients

From June 2017 to June 2018, patients who were suspected of FD were admitted to the Department of Gastroenterology in Affiliated Hospital of North Sichuan Medical College. According to CONSORT guidelines, inclusion criteria were as follows: (1) patients who met Rome IV criteria [1], presented upper abdominal pain, a burning feeling in the upper stomach, postprandial fullness, and early satiety of one or more symptoms, but no evidence of organic diseases related to the above-mentioned symptoms; existence of these symptoms for at least 6 months, as well as satisfying the Rome IV criteria for at least 3 months; (2) patients aged ≥ 18-years-old; (3) patients whose level of education was primary school and above, and had a certain reading comprehension ability. Exclusion criteria were as follows: (1) patients with organic gastrointestinal diseases, including erosive esophagitis and Barrett’s esophagus, which was diagnosed by gastroscopy; (2) patients with systemic diseases, metabolic diseases, or malignant tumors diagnosed by B-scan ultrasound, blood routine, and biochemical examinations; (3) patients with irritable bowel syndrome (IBS); (4) patients with gastroesophageal reflux disease with symptoms of acidic taste in the mouth, regurgitation, and heartburn; (5) having a history of abdominal surgery, such as cholecystectomy, intestinal resection, hysterectomy, or appendectomy; (6) patients who had recently ingested anticholinergic drugs, antispasmodic and analgesic drugs, hormones, and nonsteroidal and anti-inflammatory drugs; patients whose upper abdominal symptoms disappeared after eradicating *H. pylori* infection; (7) pregnant and lactating women; or (8) patients with psychosis and serious somatic diseases (anxiety and depression). The sample size was 395 patients in a developed IBS-specific somatization scale [27]. A minimum sample size of 300 patients achieved a 95% confidence interval to equally split chance (50/50), according to the formula, wherein 350 cases were studied [41]. Herein, we used a sample size of more than 400 patients. While developing PHQ-8, FD patients accompanied with anxiety and depression were excluded to avoid the interaction effects of anxiety, depression, and somatization disorders. However, previous studies showed that anxiety, depression, and somatization had mutual effects [21, 33]. Therefore, in the clinical application of the developed PHQ-8, FD patients with anxiety and depression could be included. All the patients signed the written informed consent form.

### Pool items for the development of the PHQ-8

According to SSS-8, PHQ-15 items and the 25 most common clinical symptoms of somatization disorder were involved in the pool items: 1. back pain; 2. pain in arms, legs, or joints; 3. dysmenorrhea or menstrual other discomfort; 4. headaches; 5. chest pain; 6. shortness of breath; 7. dizziness; 8. fainting; 9. palpitation; 10. sexual life pain or other discomfort; 11. feeling tired or having low energy; 12. insomnia or other sleep problems; 13. numbness/stinging; 14. fatigue; 15. throat discomfort (foreign body sensation/dryness/pain); 16. dry mouth; 17. excessive sweat; 18. hand/foot heavy feeling; 19. a burst of cold/fever; 20. memory loss/forgetfulness; 21. urinary frequency; 22. urinary pain/dysuria; 23. blurred vision; 24. neck and shoulder pain; 25. muscle pain. Interestingly, 1–12 items were included in the original PHQ-15 [35], in which each item was scored by three-level as 0 (“not bothered at all”), 1 (“slightly bothered”), or 2 (“remarkably bothered”). All FD patients completed a questionnaire involving 25 items by self. The items of the developed PHQ-8 were completed by discrete degree method, correlation coefficient method, factor analysis method, and Cronbach’s α.

All enrolled patients filled in the PHQ-15, the SSS-8, the Generalized Anxiety Disorder-7 (GAD-7) [42] for anxiety, the Patient Health Questionnaire (PHQ-9) [43] for depression, the DSS [33], and the Nepean Dyspepsia Index-Short Form (NDI) [44] for QoL.

### Items were selected by the four methods for the development of the PHQ-8

Discrete degree method: Since the low degree of dispersion of the selected item revealed poor ability of evaluation, the items with high degree of dispersion were selected, and the degree of dispersion was measured by the standard deviation (SD) of scores of items, in which exclusion of the SD of the items was defined as < 0.5 [45].

Correlation coefficient method: An item with a correlation coefficient > 0.3 was selected according to Cohen’s criteria [39].

Factor analysis with the principal component method: The common factor was extracted according to the feature root > 1, and the variance was maximized by orthogonal rotation to select an item with a factor load > 0.4. The Kaiser–Meyer–Olkin (KMO) and Bartlett’s spherical tests were performed. The value of KMO < 0.5 is unsuitable for factor analysis, and in Bartlett’s spherical test, *P* < 0.01 can negate the zero hypothesis that the correlation matrix is a unit matrix, i.e., a significant correlation was established between the variables [46].

Cronbach’s α coefficient: The items were screened for internal consistency, and the Cronbach’s α coefficient of the initial total scale was calculated. If the Cronbach’s α coefficient increased after the deletion of an item, it can be concluded that the existence of an item may reduce the internal consistency [47].

### DSS

The DSS score was calculated as the sum of all eight symptoms: postprandial fullness, early satiation, epigastric pain, burning, bloating, belching, nausea, and vomiting by a 3-point scale (absent, mild, moderate, severe) [33]. The correlation among the DSS, PHQ-8, and SSS-8 was evaluated, and the effects of somatization, anxiety, and depression disorders on DSS were analyzed.

### Evaluation of psychological disorder

The PHQ-15 questionnaire was designed for somatization disorder, in which the severity of 15 symptoms was rated for the last 4 weeks by three-level as 0 (“not bothered at all”), 1 (“slightly bothered”), or 2 (“remarkably bothered”) [34]. The SSS-8, composed of 8 somatic symptoms, was also applied by three-level as 0 (“not bothered at all”), 1 (“slightly bothered”), or 2 (“remarkably bothered”) [38]. The Generalized Anxiety Disorder-7 (GAD-7) was utilized to measure the severity of GAD and diagnosis comorbidity in GAD (total score > 10-point) [42]. The PHQ-9 was employed to identify major depressive disorder (total score > 10-point), and nine items were rated on a 3-point Likert scale (0–3) [43].

### Measurement of disease-specific QoL

The Short-Form Nepean Dyspepsia Index (SF-NDI) was used to measure FD patients’ QoL: inference with work/study, tension, inference with daily activities, disputation to daily eating/drinking, knowledge toward/control over disease symptoms over the past 2 weeks. Each item was assessed on a 5-point scale from 0 (not at all), 1 (slightly), 2 (moderately), 3 (remarkably) to 4 (extremely) [44]. The correlations among the SF-NDI, the PHQ-8, and the SSS-8 as well as the effects of somatization, depression, and anxiety disorders on FD patients’ QoL were assessed.

### Statistical analysis

SPSS 24.0 software (IBM, Armonk, NY, USA) was utilized to carry out statistical analysis. Quantitative data were expressed as mean ± SD, t-test was used for making comparison between two groups, and one-way analysis of variance (ANOVA) was used for comparing more than two groups. The qualitative data were presented by rate, and chi-square test was used for making comparison. Moreover, AMOS 22.0 software was applied for confirmatory factor analysis. Pearson’s correlation coefficient was used for correlation analysis. Additionally, factors influencing NDI, such as somatization, anxiety, and depression, were analyzed by linear regression analysis; *P* < 0.05 was considered statistically significant.

## Results

### Demographic characteristics and somatization scores of FD patients

Herein, 612 patients were diagnosed with FD according to Rome IV criteria. 17.3% (106/612) of the patients were identified with anxiety disorders, 13.6% (83/612) had comorbidity of depression, and 7.8% (48/612) were diagnosed with comorbidity of anxiety and depression. Among 471 FD patients without comorbidity of anxiety and depression, 63.5% (299/471) and 36.5% (172/471) of the patients were female and male, respectively. The proportion of FD patients’ level of education for primary school, middle school, high school, college or above was 22.7% (107/471), 47.3% (223/471), 14.4% (68/471), and 15.5% (73/471), respectively. The mean age of the patients was 43.4 ± 10.3 (range 18–67)-years-old. Moreover, 20.6% (97/471) of the patients were diagnosed with EPS, 31.0% (146/471) had PDS, and 48.4% (228/471) showed both EPS and PDS. The FD patients’ demographic characteristics used for PHQ-8 and the SSS-8 are shown in Table 1.

**Table 1:** Demographic characteristics of 471 FD patients versus scores of two somatization scales (mean ± SD)

	PHQ-8	t/F	*P* value	SSS-8	t/F	*P* value
Gender		2.484	0.013		3.322	0.001
Male (n = 172)	4.32 ± 2.78			4.30 ± 2.29		
Female (n = 299)	4.97 ± 2.65			5.04 ± 2.39		
Age (years)		1.439	0.231		1.987	0.115
18–29 (n = 45)	3.96 ± 2.49			3.98 ± 2.03		
30–44 (n = 201)	4.78 ± 2.61			4.84 ± 2.33		
45–59 (n = 211)	4.87 ± 2.85			4.90 ± 2.48		
≥ 60 (n = 14)	4.57 ± 2.62			4.50 ± 2.47		
Level of education		1.669	0.173		2.428	0.065
Primary school (n = 107)	5.15 ± 2.96			5.25 ± 2.76		
Junior high school (n = 223)	4.46 ± 2.71			4.54 ± 2.24		
High school (n = 68)	4.87 ± 2.63			4.96 ± 2.48		
College or above (n = 73)	4.82 ± 2.38			4.62 ± 2.03		

*FD* functional dyspepsia, *PHQ-8* Patient Health Questionnaire-8, SSS-8 Somatic Symptom Scale-8

Among the scores evaluated by these two scales, a significant difference was found in gender (*P* < 0.05). However, no significant differences were detected between different ages and levels of education (*P* < 0.05).

### Items used for developing the PHQ-8 were selected by the four methods

Using discrete degree method, chest pain, shortness of breath, fainting, palpitation, sexual life pain or other discomfort, numbness/tingling, excessive sweat, heavy hands/foot, a bursts of cold/fever, memory loss/forgetfulness, urinary frequency, urinary pain/dysuria, blurred vision, neck and shoulder pain, and muscle pain were removed since the SD of the items was defined as < 0.5.

Using correlation coefficient method, fainting (r = 0.090), dysmenorrhea or menstrual other discomfort (r = 0.243), excessive sweat (r = 0.296), and urinary pain /dysuria (r = 0.260) were removed.

According to factor analysis method, the KMO value for the initial scale was 0.668, and the value of Bartlett’s spherical test was 366.894 (*P* < 0.01), which was appropriate for factor analysis, and indicated that each item had a factor loading of > 0.4 in the dimension except for headaches and chest pain.

Cronbach’s α coefficient method showed that the α coefficient increased after excluding fainting and urinary pain/dysuria.

The screened items of the developed PHQ-8 are presented in Table 2, including back pain, pain in arms, legs, or joints, dizziness, fatigue, dry mouth, feeling tired or having low energy, insomnia or other sleep problems, and throat discomfort.

**Table 2:** PHQ-8 developed by screening 25 items using four methods

Items	Degree of dispersion	Correlation coefficient	Factor analysis	Cronbach’s α coefficient
Back pain	0.679	0.427	0.722	0.746
Pain in arms, legs, or joints	0.557	0.403	0.673	0.746
Dysmenorrhea or menstrual other discomfort	0.464(×)	0.243(×)	0.617	0.754(×)
Headaches	0.577	0.403	0.380(×)	0.746
Chest pain	0.496(×)	0.395	0.362(×)	0.745
Shortness of breath	0.497(×)	0.448	0.427	0.742
Dizziness	0.581	0.490	0.650	0.739
Fainting	0.112(×)	0.090(×)	0.609	0.755(×)
Palpitation	0.480(×)	0.386	0.514	0.746
Sexual pain or other discomfort	0.294(×)	0.313	0.511	0.749
Feeling tired or having low energy	0.687	0.543	0.731	0.735
Insomnia or trouble sleeping	0.694	0.378	0.623	0.751
Numbness/tingling	0.470(×)	0.341	0.731	0.749
Fatigue	0.628	0.509	0.780	0.738
Throat discomfort	0.763	0.457	0.434	0.746
Dry mouth	0.674	0.375	0.636	0.751
Excessive sweat	0.466(×)	0.296(×)	0.757	0.751
Hand/foot heavy feeling	0.364(×)	0.322	0.409	0.749
A burst of cold/fever	0.447(×)	0.464	0.505	0.741
Memory loss/forgetfulness	0.493(×)	0.415	0.724	0.744
Urinary frequency	0.449(×)	0.364	0.717	0.747
Urinary pain/dysuria	0.242(×)	0.260(×)	0.740	0.751
Blurred vision	0.419(×)	0.309	0.739	0.750
Neck and shoulder pain	0.487(×)	0.382	0.645	0.746
Muscle pain	0.374(×)	0.396	0.729	0.745

*PHQ-8* Patient Health Questionnaire-8, × indicated that the item was removed

### Reliability analysis of the developed PHQ-8 and the SSS-8

#### Intrinsic reliability analysis

Cronbach’s α is a measure of the internal consistency or reliability. The test is utilized to examine the consistency and stability of the questionnaires. Hence, the Cronbach’s α was applied to ascertain whether the items were reliable in measuring the same dimension. It is speculated that when the Cronbach’s α coefficient is > 0.7, the reliability is satisfactory [48]. The Cronbach α coefficient of the developed PHQ-8 and the SSS-8 was 0.601 and 0.553, respectively. The correlation coefficient between each item and the total score was 0.426–0.652 and 0.359–0.573, respectively.

#### Criterion validity

The criterion validity unveiled that the correlation coefficient between the total score of the PHQ-8 and the SSS-8 and the PHQ-15 was (r = 0.739, *P* < 0.001) and (r = 0.835, *P* < 0.001), respectively. As shown in Table 3, the developed PHQ-8 outperformed the SSS-8.

**Table 3:** Correlation analysis between somatization and quality of life of FD patients

Variable	FD (n = 471)	PDS (n = 146)	EPS (n = 97)	Overlap (n = 228)
*PHQ-8*				
r	0.309	0.281	0.190	0.360
P	< 0.001	0.001	0.063	< 0.001
*SSS-8*				
R	0.281	0.236	0.197	0.317
*P*	< 0.001	0.004	0.053	< 0.001

*FD* functional dyspepsia, *PDS* postprandial distress syndrome, *EPS* epigastric pain syndrome, *PHQ-8* Patient Health Questionnaire-8, *SSS-8* Somatic Symptom Scale-8

### Structural validity analysis for the developed PHQ-8 and the SSS-8

Exploratory factor analysis: The values of developed PHQ-8 were 0.668 and 366.894, (*P* < 0.01) in KMO and Bartlett’s spherical tests, respectively, while those for the SSS-8 were 0.680 and 236.445 (*P* < 0.01), respectively. The exploratory factor analysis was carried out on the scale, and the principal component method was used to maximize the orthogonal rotation through the covariance matrix and the variance, in which the common factor was extracted using the Kaiser criterion (Eigenvalue > 1). Additionally, 3 common factors of the developed PHQ-8 were extracted, and the cumulative contribution rate was 55.103%. The range of factor loading was 0.482–0.802, which was higher than the minimum standard of structural validity test = 0.4 [46, 48]. In addition, three common factors for the SSS-8 were extracted, and the cumulative contribution rate was 51.666%. The range of factor loading was 0.353–0.881. All coefficients related to factor loading for PHQ-8 and SSS-8 are summarized in Tables 4 and 5.

**Table 4:** Exploratory factor analysis for the developed PHQ-8

Items	Factor 1	Factor 2	Factor 3
Feeling tired or having low energy	0.802		
Fatigue	0.769		
Insomnia or trouble sleeping	0.525		
Dizziness	0.482		
Back pain		0.797	
Pain in arms, legs, or joints		0.719	
Throat discomfort			0.728
Dry mouth			0.706

*PHQ-8* Patient Health Questionnaire-8

**Table 5:** Exploratory factor analysis for the SSS-8

Items	Factor 1	Factor 2	Factor 3
Back pain	0.713		
Pain in arms, legs, or joints	0.695		
Headaches	0.495		
Chest pain or shortness of breath	0.494		
Insomnia or trouble sleeping		0.806	
Feeling tired or having low energy		0.767	
Stomach or bowel problems			0.881
Dizziness			0.353

*SSS-8* Somatic Symptom Scale-8

Confirmatory factor analysis: The model utilizes the values of chi-squared values (*χ*^2^), root mean square residual (RMR), root mean square error of approximation (RMSEA), goodness-of-fit index (GFI), adjusted GFI (AGFI), comparative fitting index (CFI), Tucker–Lewis index (TLI), normed fit index (NFI), and other indicators to evaluate the fitting effect of the model. The small value of χ^2^ and GFI, AGFI, CFI, TLI, NFI > 0.9 indicated that the model fitted well; the closer to the “l,” the better. Moreover, RMR and RMSEA < 0.05 indicated that the model fitted well, the closer to “0” fit, the better. The developed PHQ-8 and the SSS-8 were separately assessed by exploratory factor analysis, and three common factors were extracted. Two scales confirmatory factor analysis presented a good three-factor model fit (Table 6).

**Table 6:** Results of confirmatory factor analysis of the developed PHQ-8 and the SSS-8

Scale	Model	*χ*^2^	df	RMR	RMSEA	GFI	AGFI	CFI	TLI	NFI
PHQ-8	3 factors	31.247	17	0.016	0.042	0.984	0.967	0.958	0.931	0.915
SSS-8	3 factors	17.251	17	0.010	0.007	0.991	0.981	0.998	0.997	0.927

*PHQ-8* Patient Health Questionnaire-8, *SSS-8* Somatic Symptom Scale-8, *RMR* root mean square residual, *RMSEA* root mean square error of approximation, *GFI* goodness-of-fit index, *AGFI* adjusted goodness-of-fit index, *CFI* comparative fit index, *TLI* Tucker–Lewis index, *NFI* normed fit index

### Correlation analysis of anxiety, depression, somatization, and the DSS for the developed PHQ-8 and SSS-8

Somatization was evaluated by the developed PHQ-8 and the SSS-8. Correlation analysis showed that anxiety, depression, and somatization were positively correlated with DSS (Table 7). The correlation coefficient between DSS and somatization assessed by the SSS-8 was higher than that of the developed PHQ-8.

**Table 7:** Correlation analysis of somatization, anxiety, depression, and the dyspepsia symptom severity

Variable	FD (n = 612)	PDS (n = 175)	EPS (n = 112)	Overlap (n = 325)
*Anxiety*				
R	0.223	0.231	0.147	0.232
P	< 0.001	< 0.001	< 0.001	< 0.001
*Depression*				
R	0.323	0.296	0.322	0.333
P	< 0.001	< 0.001	< 0.001	< 0.001
*Somatization PHQ-8*				
R	0.374	0.396	0.293	0.346
P	< 0.001	< 0.001	< 0.001	< 0.001
*Somatization SSS-8*				
R	0.399	0.395	0.281	0.362
P	< 0.001	< 0.001	< 0.001	< 0.001

*FD* functional dyspepsia, *PDS* postprandial distress syndrome, *EPS* epigastric pain syndrome, *PHQ-8* Patient Health Questionnaire-8, *SSS-8* Somatic Symptom Scale-8

### Linear regression analysis of the effects of anxiety, depression, somatization on the DSS

Somatization, anxiety, and depression were taken as independent variables, while the DSS was taken as a dependent variable, and linear regression analysis was conducted by the backward elimination method. After adjusting for factors, such as gender, age, type of FD, level of education, and employment situation, depression and somatization were found as factors influencing the DSS (Table 8). The adjusted R^2^ for the PHQ-8 and the SSS-8 was 0.263 and 0.263, respectively (all *P* < 0.001). Also, the role of somatization was found to be more significant than that of depression. The standardized β for the SSS-8 seemed to be higher than that for the PHQ-8.

**Table 8:** Effects of somatization, anxiety, and depression on the dyspepsia symptom severity

Variable	Unstandardized β	Standardized β	t-value	*P* value
Depression	0.144	0.176	3.911	< 0.001
Somatization PHQ-8	0.287	0.256	6.460	< 0.001
Depression	0.154	0.188	4.218	< 0.001
Somatization SSS-8	0.206	0.257	6.431	< 0.001

*PHQ-8* Patient Health Questionnaire-8, *SSS-8* Somatic Symptom Scale-8

### Correlation analysis of anxiety, depression, somatization, and QoL

Correlation analysis showed that somatization, anxiety and depression were positively correlated with the NDI (Table 9). Compared to the SSS-8, the correlation coefficient between NDI and somatization for the developed PHQ-8 was higher.

**Table 9:** Correlation analysis of somatization, anxiety, depression and the Nepean Dyspepsia Index

Variable	FD (n = 612)	PDS (n = 175)	EPS (n = 112)	Overlap (n = 325)
*Anxiety*				
R	0.327	0.258	0.268	0.367
P	< 0.001	< 0.001	< 0.001	< 0.001
*Depression*				
R	0.354	0.266	0.304	0.394
P	< 0.001	< 0.001	< 0.001	< 0.001
*Somatization PHQ-8*				
R	0.404	0.325	0.309	0.446
P	< 0.001	< 0.001	< 0.001	< 0.001
*Somatization SSS-8*				
R	0.389	0.285	0.345	0.418
*P*	< 0.001	< 0.001	< 0.001	< 0.001

*PHQ-8* Patient Health Questionnaire-8, *SSS-8* Somatic Symptom Scale-8

### Linear regression analysis of the effects of anxiety, depression, and somatization on QoL

The NDI for QoL was taken as a dependent variable, and somatization, anxiety, and depression were independent variables. The linear regression analysis was undertaken by backward elimination method. After adjusting for factors, such as gender, age, type of FD, level of education, and employment, situation, somatization, anxiety, and depression were noted as factors influencing the QoL (Table 10). The adjusted R^2^ for the PHQ-8 and the SSS-8 was 0.224 and 0.236, respectively (all *P* < 0.001). Somatization appeared to be more important than anxiety and depression. The standardized β for the PHQ-8 was greater than that for the SSS-8.

**Table 10:** The effects of somatization, anxiety, and depression on the Nepean Dyspepsia Index

Variable	Unstandardized β	Standardized β	t-value	*P* value
Anxiety	0.567	0.163	3.784	< 0.001
Depression	0.490	0.136	3.986	0.003
Somatization (PHQ-8)	1.330	0.270	6.638	< 0.001
Anxiety	0.541	0.156	3.580	< 0.001
Depression	0.557	0.155	3.416	0.001
Somatization (SSS-8)	0.883	0.250	6.311	< 0.001

*PHQ-8* Patient Health Questionnaire-8, *SSS-8* Somatic Symptom Scale-8

## Discussion

The FD is the result of the interaction of physiological function and psychosocial factors through brain-gut axis changes [2, 5]. Somatization in psychosocial factors influences the health-related QoL after controlling the symptoms of anxiety and depression disorders in FD patients [30, 49]. These factors may lead to the deterioration of symptoms by aggravating anxiety and depression, resulting in repeated medical treatment and economic burden [33]. Somatization symptoms are extensively assessed by PHQ-15, possessing high reliability and validity not only in general population, but also in patient population [34, 50].

However, the assessment of somatization symptoms by the PHQ-15 exhibits several limitations. First, the PHQ-15 is not a specific scale for FD patients, which has three items overlapping with gastrointestinal symptoms. Secondly, there is a menstrual problem which is not applicable to menopausal women and may cause differences in gender analysis. Thirdly, due to cultural differences, patients assessed by the PHQ-15 have a poor compliance with questions related to sexual issues. Fourthly, the relative incidence of syncopal items is low [35]. Finally, the majority of patients with somatic symptoms are often examined in a general outpatient clinic without non-psychiatric doctors, which might result in less satisfactory results. Hence, it is highly essential to use a simple tool to assess the FD patients’ somatization symptoms and severity, which is conducive to doctors as they can advise the patients about reliable therapeutic choices.

Therefore, the SSS-8 has been used for fast assessment of somatization symptoms and its severity, avoiding differences in gender caused by menstrual abnormalities, sexual life problems caused by cultural differences, and low incidence of syncopal items [38], while it is a non-specific scale for FD patients and overlaps with gastrointestinal symptoms in the clinical application. In addition, the SSS-8 did not include FD patients’ typical symptoms, such as throat pain, which might lead to neglect those symptoms by clinicians.

Hence, due to limitations of the SSS-8, in the present study, the PHQ-8 was developed by screening 25 items related to somatization. Gierk et al. [40] used confirmatory factor analysis to analyze the validity of the SSS-8, and the results showed that the 3-factor model outperformed the 1-factor model. Similarly, in the current study, we used a 3-factor model for confirmatory analysis of the PHQ-8 and the SSS-8. The 3-factor model fitted for the SSS-8 showed a superior performance than that of the PHQ-8.

However, the developed PHQ-8 was superior to the SSS-8 for FD patients as follows: firstly, the PHQ-8 is a specific scale for FD patients; the PHQ-8 includes typical symptoms of somatization, such as throat pain, which has no overlap with gastrointestinal symptoms. Secondly, in the PHQ-8, each item is significantly correlated with the total score, reflecting a higher reliability of the PHQ-8 than that of the SSS-8. Thirdly, the PHQ-8 may be superior than the SSS-8 in the internal consistency. Fourthly, the PHQ-8 has a higher cumulative contribution rate than the SSS-8 in the exploratory factor analysis. Finally, the PHQ-8 is markedly correlated with NDI as compared to SSS-8, when NDI is used as a classic QoL assessment scale in the criterion validity.

The developed PHQ-8 was further evaluated in order to compare with the SSS-8 for 612 FD patients, including anxiety or depression, because previous studies showed that anxiety, depression, and somatization had mutual effects [21, 33]. The results of the present study unveiled that the SSS-8 might play a significant role in the DSS than the PHQ-8 because of the fact that the SSS-8 overlaps with gastrointestinal symptoms. However, somatization assessed by the PHQ-8 may have a greater influence on QoL than that of the SSS-8 in FD patients.

Nevertheless, the present has some limitations. Firstly, the patients with FD came from tertiary hospitals. These patients may have severe symptoms, limiting the prevalence of other people with FD. However, whether other FD patients from the community can achieve the same conclusion requires further investigation. Secondly, this study mainly analyzed and evaluated specific somatization symptoms of FD patients by the developed PHQ-8; the cohort did not involve healthy individuals and patients with other diseases (irritable bowel syndrome). Thirdly, the PHQ-8 was not tested for retesting reliability in all the cases; thus, the reliability of the scales needs to be further confirmed. Fourthly, similar to the SSS-8, the PHQ-8 was used to assess the severity of somatoform disorders [34]. Finally, the data obtained via questionnaires were often abnormally distributed, while those similar to normally distributed data were reported in previous large-sample studies [33, 44]. Accordingly, further studies with large sample size are required to improve the rationality of the developed PHQ-8.

In presence of the above-mentioned limitations, the results of the present study showed that the PHQ-8 developed for evaluating somatization and QoL outperformed the SSS-8 in FD patients not only without but also with anxiety and depression. All patients self-completed the PHQ-8. Somatization may be a major factor influencing DSS and NDI as compared to anxiety and depression, as well as *H. pylori* infection, gastric sensitivity, gastric accommodation, and gastric emptying in FD patients [21, 34]. Somatization is mainly treated by antidepressant drugs, which were often used to treat functional dyspepsia in patients who do not exhibit the effects of combined prokinetic and proton pump inhibitor therapy [34, 51]. Therefore, evaluating somatization is essential in the study of FD patients; also, PHQ-8 might be applied to assess the effects of antidepressant drugs to treat functional dyspepsia. Therefore, PHQ-8 might be valuable in assessing somatization using SSS-8 in the future FD-based studies.

## Conclusions

The PHQ-15 and the SSS-8 have overlap in gastrointestinal symptoms in FD patients, and the SSS-8 was recommended to be a replacement for the PHQ-15 in a short period of time. The developed PHQ-8 without having overlap in gastrointestinal symptoms showed to have satisfactory reliability and validity. Somatization assessed by the developed PHQ-8 may have a greater impact on QoL of FD patients compared with the SSS-8. Our findings suggested that the developed PHQ-8 may be more effective for evaluating the influences of somatization on QoL of FD patients rather than SSS-8, which may be helpful to further verify the efficacy of the developed PHQ-8 to improve a more reliable assessment for the effects of somatization on FD patients.

## Data Availability

The data that support the findings of this study are available from the corresponding author upon a reasonable request.

## Abbreviations

FD: Functional dyspepsia
SSS-8: Somatic Symptom Scale-8
PHQ-8: Patient Health Questionnaire-8
QoL: Quality of life
NDI: Nepean Dyspepsia Index
RMR: Root mean square residual
RMSEA: Root mean square error of approximation
GFI: Goodness-of-fit index
AGFI: Adjusted goodness-of-fit index
CFI: Comparative fit index
TLI: Tucker–Lewis index
NFI: Normed fit index
EPS: Epigastric pain syndrome
PDS: Postprandial distress syndrome
PHQ-15: Patient Health Questionnaire-15
PHQ-12: Patient Health Questionnaire-12
GAD-7: Generalized Anxiety Disorder-7
PHQ-9: Patient Health Questionnaire-9
DSS: Dyspepsia symptom severity
